# New Strategies in Preventing Inflammatory and/or Oxidative Stress-Induced Damages in Ischemia–Reperfusion Injury (2nd Edition)

**DOI:** 10.3390/antiox14111329

**Published:** 2025-11-05

**Authors:** Borja Herrero de la Parte, Ignacio García-Alonso, Ana Alonso-Varona

**Affiliations:** 1 Department of Surgery, Radiology and Physical Medicine, Faculty of Medicine and Nursing, University of the Basque Country (UPV/EHU), 48940 Leioa, Spain; 2 Department of Cell Biology and Histology, Faculty of Medicine and Nursing, University of the Basque Country (UPV/EHU), 48940 Leioa, Spain

Ischemia–reperfusion injury remains one of the most fascinating and challenging paradoxes in modern biomedicine. The restoration of blood flow, which is essential for preserving tissue viability, triggers a cascade of biochemical events that often exacerbate the initial damage. The overproduction of reactive oxygen species, mitochondrial dysfunction, the activation of inflammatory pathways, and endothelial dysfunction form a complex network that compromises organ function. Despite decades of research, effective prevention of this syndrome remains a challenge in many clinical settings, from transplantation and cardiovascular surgery to neurovascular events and intestinal ischemia.

The first edition of this Special Issue of *Antioxidants* served to highlight the central role of oxidative stress and antioxidant therapies in ischemia–reperfusion injury, as well as to bring together promising experimental strategies aimed at mitigating its effects. In this second edition, the focus broadens to a more integrative view, in which redox, immunological, and metabolic mechanisms are conceived as an interdependent and dynamic system. This perspective allows us to explore new therapeutic approaches capable of acting synergistically on the different levels of the lesion cascade.

The articles collected in this edition offer an updated and diverse view of the problem. Among the experimental works, the study by Sánchez-Bonet (contribution 1) and colleagues stands out, demonstrating the protective potential of a *Fucus vesiculosus* extract against intestinal ischemia, restoring the activity of antioxidant enzymes and reducing mucosal inflammation. In the cerebral sphere, Romaus-Sanjurjo et al. (contribution 2) show how the administration of low doses of TWEAK can mobilize endothelial progenitors and promote vascular repair after intracerebral hemorrhage, while Sigdel et al. (contribution 3) describe the beneficial effect of extracellular vesicles released after physical exercise on cerebral endothelial cells exposed to hypoxia and angiotensin II, opening up the door to non-pharmacological interventions based on “cell preparation”.

At the heart of this research, two complementary approaches highlight the relevance of molecular and epigenetic modulation of oxidative stress. Cucinotta et al. (contribution 4) document the cardioprotective effect of the prolyl endopeptidase inhibitor KYP-2047, which attenuates inflammatory infiltration and apoptosis after myocardial reperfusion. Park et al. (contribution 5) demonstrated that the inhibition of microRNA-25 restores Sestrin3 expression and reduces oxidative burden, consolidating the role of microRNAs as modulators of redox balance.

In the field of experimental nephrology, the work of Jang et al. (contribution 6) provides an innovative insight by demonstrating that intermittent fasting mitigates epithelial–mesenchymal transition and renal fibrosis after ischemia, pointing to the relevance of metabolism and caloric restriction as modulators of the progression from acute injury to chronic disease. Complementarily, Troise and his team review (contribution 7) the involvement of the complement system in renal graft dysfunction after transplantation and propose the targeted inhibition of its components as an effective tool to reduce reperfusion injury and improve early function of the transplanted organ.

The two review articles included in this Special Issue offer a conceptual framework that broadens our understanding of IRI. Dery et al. (contribution 8) analyze the feedback loops between oxidative stress and the immune response in the liver, highlighting the relevance of redox metabolites and endoplasmic reticulum stress as mediators of inflammatory activation, while Dhalla et al. (contribution 9) provide a comprehensive update on the mechanisms of myocardial reperfusion injury, from mitochondrial ROS generation to impaired calcium handling, and review the most promising antioxidant therapeutic approaches.

Finally, Berzenji et al. (contribution 10) provide a review focusing on pulmonary ischemia–reperfusion injury in the context of transplantation. This review summarizes the pathophysiology of primary graft dysfunction and the oxidative and inflammatory mechanisms involved. It also discusses advances in ex vivo lung preservation and perfusion techniques. This review emphasizes the importance of integrated protection strategies—pharmacological, mechanical, and physiological—to optimize lung viability and reduce postoperative complications.

Together, these works reflect the maturity of a field that has evolved from the simple neutralization of free radicals to a much more sophisticated understanding of the interconnected processes that underlie reperfusion injury. Recent research clearly indicates that oxidative, inflammatory, and metabolic mechanisms do not act in isolation but rather as part of a complex regulatory network. Hence, emerging strategies tend to combine approaches: mitochondria-targeted antioxidants, complement modulators, microRNA inhibitors, or redox-sensitive controlled-release systems. Therapeutic nanotechnology and gene therapies appear to be promising tools for achieving precise, localized, and safe intervention.

At the same time, the studies included in this edition remind us of the importance of biological and behavioral factors that had traditionally been overlooked in antioxidant therapy. The influence of physical exercise, diet, and intermittent fasting on the cellular response to ischemic stress shows that the concept of “physiological conditioning” can be as relevant as pharmacology. This holistic view, in which the body prepares itself to resist oxidative stress, opens up a line of research that transcends the classic boundaries between prevention and treatment.

The future of ischemia–reperfusion research lies in integrating these approaches and transferring them to the clinical setting with rigorous and translational criteria. It will be essential to define robust biomarkers that allow for the optimal therapeutic window to be identified, rational combinations of treatments to be selected, and their long-term functional impact to be evaluated. IRI is not a single, uniform process: its manifestation varies according to the organ, time, and metabolic context. Consequently, therapeutic strategies must be equally specific and dynamic.

The second edition of this Special Issue of *Antioxidants* reflects the vitality of a field undergoing continuous transformation. The papers collected here not only expand our knowledge of the mechanisms of damage and defense but also point to a paradigm shift in the approach to ischemia–reperfusion: from isolated antioxidant therapy to the integrated modulation of redox, immune, and metabolic responses. We trust that this collection will inspire new lines of research, foster interdisciplinary collaboration, and contribute to bringing the advances currently being developed in the laboratory closer to clinical practice.

